# Fucoidan from *Ascophyllum* *nodosum* Suppresses Postprandial Hyperglycemia by Inhibiting Na^+^/Glucose Cotransporter 1 Activity

**DOI:** 10.3390/md18090485

**Published:** 2020-09-22

**Authors:** Xindi Shan, Xueliang Wang, Hao Jiang, Chao Cai, Jiejie Hao, Guangli Yu

**Affiliations:** 1 Key Laboratory of Marine Drugs of Ministry of Education, Shandong Provincial Key Laboratory of Glycoscience and Glycotechnology, School of Medicine and Pharmacy, Ocean University of China, Qingdao 266003, China; shanxindi@hotmail.com (X.S.); wangsun13141314@126.com (X.W.); haojiang@ouc.edu.cn (H.J.); caic@ouc.edu.cn (C.C.); 2 Laboratory for Marine Drugs and Bioproducts, Pilot National Laboratory for Marine Science and Technology (Qingdao), Qingdao 266237, China

**Keywords:** fucoidan from *Ascophyllumnodosum*, postprandial hyperglycemia, in vitro and in vivo evaluation, SGLT1

## Abstract

We previously demonstrated that fucoidan with a type II structure inhibited postprandial hyperglycemia by suppressing glucose uptake, but the mechanism remains elusive. Here, we aimed to assess whether the effect of glucose absorption inhibition was related to the basic structure of fucoidans and preliminarily clarified the underlying mechanism. Fucoidans with type II structure and type I structure were prepared from *Ascophyllum* *nodosum* (AnF) or *Laminaria* *japonica* (LjF) and *Kjellmaniella* *crassifolia* (KcF), respectively. The effects of various fucoidans on suppressing postprandial hyperglycemia were investigated using in vitro (Caco-2 monolayer model), semi-in vivo (everted gut sac model), and in vivo (oral glucose tolerance test, OGTT) assays. The results showed that only AnF with a type II structure, but not LjF or KcF with type I structure, could inhibit the glucose transport in the Caco-2 monolayer and everted gut sac models. A similar result was seen in the OGTT of Kunming mice and leptin receptor-deficient (db/db) mice, where only AnF could effectively inhibit glucose transport into the bloodstream. Furthermore, AnF (400 mg/kg/d) treatment decreased the fasting blood glucose, HbA1c, and fasting insulin levels, while increasing the serum glucagon-like peptide-1 (GLP-1) level in obese leptin receptor-deficient (db/db) mice. Furthermore, surface plasmon resonance (SPR) analysis revealed the specific binding of AnF to Na^+^/glucose cotransporter 1 (SGLT1), which indicated the effect of AnF on postprandial hyperglycemia could be due to its suppression on SGLT1 activity. Taken together, this study suggests that AnF with a type II structure can be a promising candidate for hyperglycemia treatment.

## 1. Introduction

Diabetes mellitus (DM) is a group of metabolic disorders that are characterized by hyperglycemia [1]. Hyperglycemia is caused by the combined action of insulin deficiency and/or insulin resistance in diabetic patients [2]. Long-term hyperglycemia is associated with microvascular complications and leads to cardiovascular disease, diabetic nephropathy, and other severe complications, which are the major causes of death in diabetic patients [3]. In the early stages of DM, the effective control and strict management of postprandial blood glucose levels are crucial for alleviating DM. The current effective ways to reduce postprandial blood glucose level are as follows: (I) inhibit the activities of digestive enzymes, such as glucosidases and α-amylase, which can produce glucose from starch and other carbohydrates [4]; (II) promote the secretion of insulin, such as by inhibiting dipeptidyl peptidase 4 (DPP-4) and elevating glucagon-like peptide 1 (GLP-1) activities [5,6]; (III) reduce glucose reabsorption in the kidney by inhibiting Na^+^/glucose cotransporter 2 (SGLT2) activity [7]; (IV) suppress the glucose absorption into the bloodstream via the small intestinal by inhibiting the function of Na^+^/glucose cotransporter 1 (SGLT1) [8].

Although there are several oral hypoglycemic agents that can reduce the blood glucose level in DM, these agents are mainly chemically synthetic, costly, and can cause severe adverse side-effects (e.g., serious hypoglycemia and kidney damage) [9,10]. Thus, the discovery of effective, safer, and affordable drugs for DM patients has attracted significant research attention. Research suggests that certain natural herbal products can exert hypoglycemic effects and are safer and easier to obtain than other synthetic chemicals [11]. Convincing evidence suggests that bioactive compounds (e.g., proteins, polyphenols, tannins, and phenolic acids) can directly affect intestinal glucose uptake by competitive inhibition of the glucose transporter SGLT1 [12,13,14]. In the small intestine, SGLT1 is expressed in the apical cell membrane constituting the brush border [15], which is of primary importance for glucose absorption from the lumen to the epithelial cells of the intestine [16,17]. Ina et al. demonstrate that rice albumin could alleviate postprandial hyperglycemia by inhibiting SGLT1 function via in vivo and in vitro assays [18]. Müller et al. confirmed that the extracts of guava leaves and fruits, which contain polysaccharides, polyphenols, and other bioactive substances, can effectively reduce intestinal glucose transport by inhibiting the function of SGLT1 in vivo [19].

An increasing number of studies have demonstrated that non-toxic biological macromolecules, especially polysaccharides, possess prominent efficacies for treating DM and other metabolic diseases [20,21,22,23,24]. In addition, Tang et al. reported that water-soluble polysaccharides from *Lycium barbarum* have a conspicuously inhibitory effect on glucose uptake in vitro [25], which may have been due to the inhibitory effect on the intestinal glucose transporter SGLT1. Fucoidan is a family of sulfated fucan predominantly existing in the cell walls of brown algae and several marine invertebrates (e.g., sea cucumbers and sea urchins) [26]. These water-soluble heteropolysaccharides are composed of various percentages of L-fucose and sulfate ester groups. Fucoidans from natural sources are usually composed of two types of chain structures, type I, with α (1→3)-linked fucose, and type II, made up of alternating α (1→3)- and α (1→4)-linked fucose molecules [27]. In recent years, fucoidans isolated from different sources have been extensively studied due to their diverse biological activities, including anticoagulant, anti-inflammatory, antivirus, antitumor, lipid-lowering, antidiabetic nephropathy, antimetabolic syndrome, and prebiotic effects [26,28]. Due to their promising therapeutic effects and availability from various kinds of cheap brown algae, an increasing number of studies have been devoted to the development and utilization of fucoidans in the fields of drugs and functional foods. Although the application prospects of fucoidans are promising, it is worth noting that the bioactivities of fucoidans are probably highly dependent on their structural properties (such as type of glycosidic linkages, molecular weight (MW), and branches) [26,29] and little attention has been devoted to determining the effects of fucoidans with various structure on attenuating postprandial hyperglycemia and its underlying mechanism.

We reported that fucoidan from *Fucus vesiculosus* (FvF) with a type II structure can significantly inhibit α-glucosidase and the glucose transport activities in the small intestine, and regulate glucose consumption and lipid metabolism via reactive oxygen species (ROS)-mediated c-Jun N-terminal kinase (JNK) and protein kinase B (Akt) signaling pathways, thus improving postprandial hyperglycemia in diabetic mice [30,31]. To explore the relationship between the inhibitory effect of glucose absorption and fucoidan, it is necessary to rule out the other factors related to antihyperglycemia. Therefore, we prepared three fucoidans as follows: fucoidan from *Ascophyllum nodosum* (AnF) with a type II structure [32] and mild inhibition of α-glucosidase [31], and fucoidans from *Laminaria japonica* (LjF) and *Kjellmaniella crassifolia* (KcF) with type I structures [33,34]. Additionally, we investigated the pharmacological effects of the above fucoidans on alleviating postprandial hyperglycemia using in vitro (Caco-2 monolayer model), semi-in vivo (everted gut sac model), and in vivo (oral glucose tolerance test (OGTT) in Kunming and leptin receptor-deficient (db/db) mice) assays. Based on previous studies [25,30], we also focused on evaluating the binding affinity of various fucoidans to SGLT1 via surface plasmon resonance (SPR). Taken together, as we have previously reported, FvF with a type II structure could alleviate the postprandial hyperglycemia. Thus, this study aimed to evaluate the effects of fucoidans with different types of fucosidic linkages on alleviating postprandial hyperglycemia and preliminarily elucidated the underlying mechanism.

## 2. Results

### 2.1. Physicochemical Properties of Fucoidans from Various Sources

The basic physicochemical properties of AnF, LjF, and KcF were determined and are summarized in Table 1. The sulfate content ranged from 22% to 28%, and the sulfate content of AnF was lower compared with that of LjF or KcF. The MWs of AnF and LjF were similar, which were both much lower than that of KcF (*p* < 0.05). According to our monosaccharide composition analysis, these three fucoidans were mainly composed of fucose, and the contents of fucose were similar in AnF and LjF, while KcF had a much higher fucose content (*p* < 0.05). The above results were consistent with that reported in previous literatures [32,33,34]. In general, the main differences between these three fucoidans were the type of glycosidic linkages, MWs, and fucose content. The bioactivities of fucoidans with various physicochemical properties can be different. Therefore, we further investigated the effects of these three fucoidans on alleviating postprandial hyperglycemia in vitro and in vivo.

### 2.2. Effect of Fucoidans on Inhibiting Glucose Transport in a Caco-2 Monolayer Cell Model

**Figure 1:**
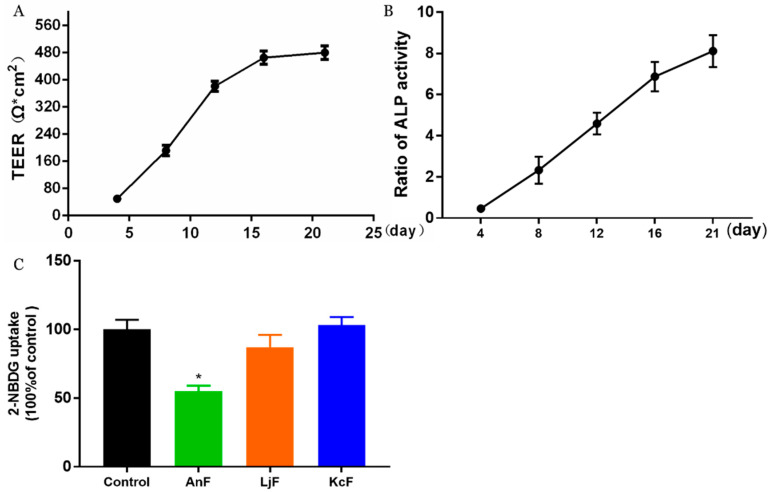
Inhibitory effects of fucoidans on 2-Deoxy-2-[(7-nitro-2,1,3-benzoxadiazol-4-yl) amino]-D-glucose (2-NBDG) transport in a Caco-2 monolayer cell model. The transepithelial electrical resistance (TEER) of Caco-2 monolayer cell model (**A**); alkaline phosphatase (ALP) activity ratio of Caco-2 monolayer cell model (**B**), calculated by the ratio of ALP activities on the apical side to the basolateral side; inhibitory effects of fucoidans on 2-NBDG transport (**C**). Control, Caco-2 monolayer cell model treated with HBSS buffer; *Ascophyllum nodosum* (AnF), Caco-2 monolayer cell model treated with 400 μg/mL of AnF; or *Laminaria japonica* (LjF): Caco-2 monolayer cell model treated with 400 μg/mL of LjF; *Kjellmaniella crassifolia* (KcF): Caco-2 monolayer cell model treated with 400 μg/mL of KcF. Data are expressed as the mean ± SEM. * *p* < 0.05, compared with the Control group.

Here, we used a Caco-2 monolayer cell model to evaluate the influences of various fucoidans on intestinal glucose transport. The Caco-2 monolayer cell model is a well-established in vitro model for studying the transport of substrates (e.g., glucose, nutrients, and drugs) through the intestine [35]. The prerequisite for the simulation of in vivo intestinal processes is the differentiation of the Caco-2 monolayer cell, which expresses a tissue-typical cell membrane and transport proteins. In addition, it has been verified that there is a high expression of endogenous SGLT1 in polarized Caco-2 cells [36]. As shown in Figure 1A, the transepithelial electrical resistance (TEER) value rose rapidly from 49 Ω*cm^2^ on the fourth day to about 476 Ω*cm^2^ after a 16-day incubation. Then, this cell monolayer model tended to be completed, and the resistance value changed little on the 21st day compared to that on the 16th day, which indicated that the cells started to differentiate. Moreover, the activities of alkaline phosphatase (ALP) on both sides of the transwell chamber were measured to judge the degree of cell differentiation and the success of the cell model [37]. As shown in Figure 1B, the ratio of ALP activities significantly increased to about 8 during the 21 days incubation, which indicated that the degree of differentiation in Caco-2 cells was high, and the cells displayed obvious polarization. The above results indicated that a Caco-2 monolayer cell model was successfully constructed, which was then used to study the effects of fucoidans on the transmembrane transport of glucose. The inhibitory activity of specific fucoidan on the transport of 2-Deoxy-2-[(7-nitro-2,1,3-benzoxadiazol-4-yl) amino]-D-glucose (2-NBDG) using the Caco-2 monolayer model is shown in Figure 1C. These results showed that only 400 μg/mL of an AnF solution could significantly inhibit the transport of 2-NBDG in this Caco-2 monolayer cell model compared with the Control group (*p* < 0.05), while the same concentration of LjF and KcF could not. Compared to the effects of AnF and LjF on 2-NBDG transport, our results indicate that the type of glycosidic linkages may play a crucial role in inhibiting glucose transport. In addition, both LjF and KcF had no marked effects on glucose transport in the Caco-2 monolayer cell model, which indicated that MW and fucose content may not play a pivotal role in that. We have previously reported that FvF could significantly reduce glucose transport in a Caco-2 monolayer cell mode [30]. In general, only AnF and FvF, with type II structures, could inhibit the glucose transport in a Caco-2 monolayer cell model, which indicated the importance of the type of glycosidic linkages in inhibiting glucose transport.

### 2.3. Effect of Fucoidans on Intestinal Glucose Uptake Using an Everted Gut Sac Model

**Figure 2:**
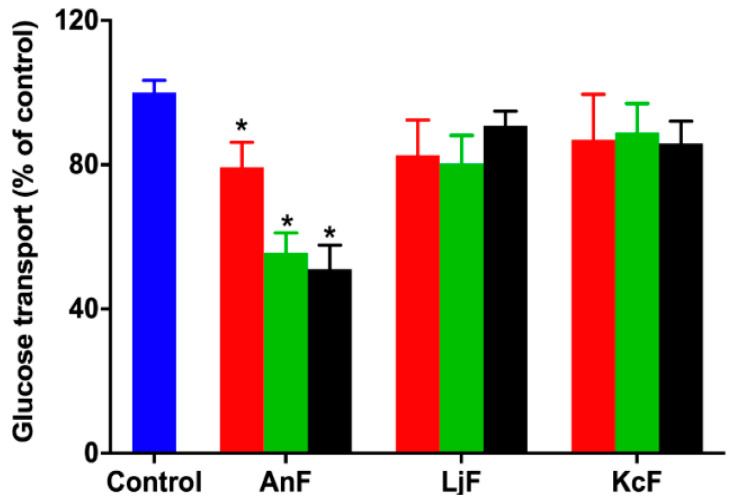
Inhibition rate of different concentrations of fucoidans on glucose uptake in an everted gut sac model. Control, the everted gut sac model treated with Krebs–Ringer bicarbonate buffer; AnF, the everted gut sac model treated with various concentrations of AnF; LjF, the everted gut sac model treated with various concentrations of LjF; KcF, the everted gut sac model treated with various concentrations of KcF. Red represents the results of treatment with 100 μg/mL of specific fucoidan; green represents the results of treatment with 400 μg/mL of specific fucoidan; black represents the result of treatment with 600 μg/mL of fucoidan. Data are expressed as the mean ± SEM. * *p* < 0.05, specific concentration of AnF treated group compared with the Control group.

Glucose can be transported from the intestinal lumen into small intestinal enterocytes by SGLT1, which is located at the apical brush border [38]. SGLT1 was originally expressed on the serous side of the intestine and was moved to the outside in an everted gut sac model. Thus, the everted gut sac model can be used as an efficient semi-in vivo tool for studying substrates and drug absorption mechanisms, as well as the role of compounds on regulating SGLT1 activity in the intestine [39,40]. Therefore, the inhibition of glucose absorption was evaluated using the everted gut sac model to explore the inhibitory effect on SGLT1 activity. As shown in Figure 2, 100 μg/mL of AnF could effectively decrease the glucose absorption compared with the Control group (*p* < 0.05), and this inhibitory effect was elevated with an increased AnF concentration, while LjF and KcF showed no significant inhibitory effect in the range from 100 μg/mL to 600 μg/mL. All in all, the above results demonstrated that only AnF with a type II structure had a marked inhibition on glucose intake in a dose-dependent manner via inhibiting SGLT1 activity, which was consistent with the results in Section 2.2. Once again, the type of glycosidic linkages was shown to play an important role in suppressing glucose absorption from the intestinal lumen.

### 2.4. Effects of Fucoidans on OGTT in Kunming Mice

**Figure 3:**
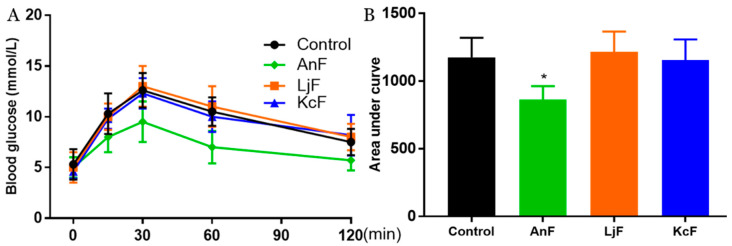
Effects of fucoidans on the oral glucose tolerance test (OGTT) in Kunming mice. Curve of OGTT (**A**) and area under curve (**B**). Control, Kunming mice gavaged with PBS; AnF, Kunming mice gavaged with 200 mg/kg of AnF; LjF: Kunming mice gavaged with 200 mg/kg of LjF; KcF: Kunming mice gavaged with 200 mg/kg of KcF. Data are expressed as the mean ± SEM. * *p* < 0.05, compared with the Control group. Six mice of each group were analyzed.

Intestinal glucose absorption is mediated by SGLT1 [41]. First, the carbohydrates in food are degraded to monosaccharides (such as glucose, galactose, etc.) through the action of various glycosidases. Next, glucose is then transported into the cells on the mucosal side by SGLT1. In addition, it has been confirmed that fucoidans have a certain inhibitory effect on α-glucosidase [42] and cannot be digested by gastric and pancreatic enzymes [43]. Therefore, the OGTT was used to explore the inhibitory effects of fucoidans on glucose absorption via inhibiting SGLT1 activity, which can avoid the influence of glucosidase. As shown in Figure 3, the administration of 200 mg/kg of AnF effectively suppressed the elevation in the postprandial blood glucose level and the areas under curve after glucose loading in Kunming mice compared with the Control group (*p* < 0.05), while LjF and KcF treatments could not. The above results verified the efficient decrease in postprandial blood glucose conferred by AnF treatment, as compared to LjF and KcF in Kunming mice, which was consistent with our results from in vitro and semi-in vivo assays (shown in Section 2.2 and Section 2.3). Thus, we further investigated and evaluated the hypoglycemic effect of AnF in db/db mice.

### 2.5. Effects of AnF on the OGTT in db/db Mice

**Figure 4:**
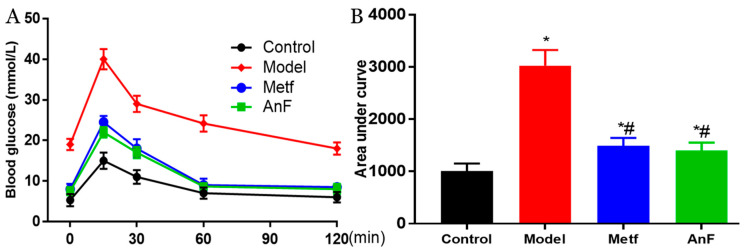
Effects of AnF on OGTT in leptin receptor-deficient (db/db) mice. Curve of OGTT (**A**) and area under curve (**B**). Control, C57BL/6J mice; Model, db/db mice; metformin (Metf), db/db mice with 200 mg/kg/d of metformin; AnF: db/db mice with 200 mg/kg/d of AnF. Data are expressed as the mean ± SEM. * *p* < 0.05, compared with the Control group; # *p* < 0.05, compared with the Model group. Six mice of each group were analyzed.

OGTT is the gold standard for DM diagnosis [44] and is used to evaluate the function of β cells and the individual ability to regulate postprandial blood glucose levels. Thus, a glucose solution was gavaged after 15 h of fasting at the end of a 4-week feeding trial in db/db mice. The AnF group showed remarkable suppression of OGTT and the area under curve in db/db mice (*p* < 0.05), which was comparable to the effects of metformin (Metf) (Figure 4). This result indicated that AnF can improve blood glucose homeostasis in mice with diabetes.

### 2.6. Effects of AnF on Body Weight in db/db Mice

**Figure 5:**
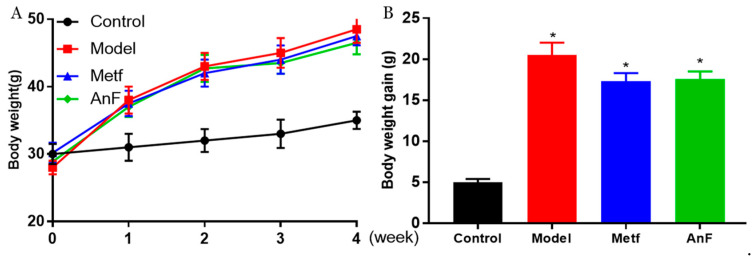
Effect of AnF on body weight change in db/db mice. Body weight change (**A**) and body weight gain (**B**). Control, C57BL/6J mice; Model, db/db mice; Metf, db/db mice with 200 mg/kg/d of metformin; AnF, db/db with 200 mg/kg/d AnF. Data are expressed as the mean ± SEM. * *p* < 0.05, compared with the Control group. Six mice of each group were analyzed.

During the 4-weeks feeding trial, db/db mice gained much more weight compared to normal C57BL/6J mice (*p* < 0.05) (Figure 5), while both AnF and metformin induced a trend of weight loss in db/db mice. In addition, another study in our lab showed that AnF significantly reduced the body weight gain of mice, which were fed with a high-fat diet [28]. The above results indicated that AnF could effectively lower the body weight in mice with DM.

### 2.7. Effects of AnF on Glucose-Insulin Homeostasis in db/db Mice

**Figure 6:**
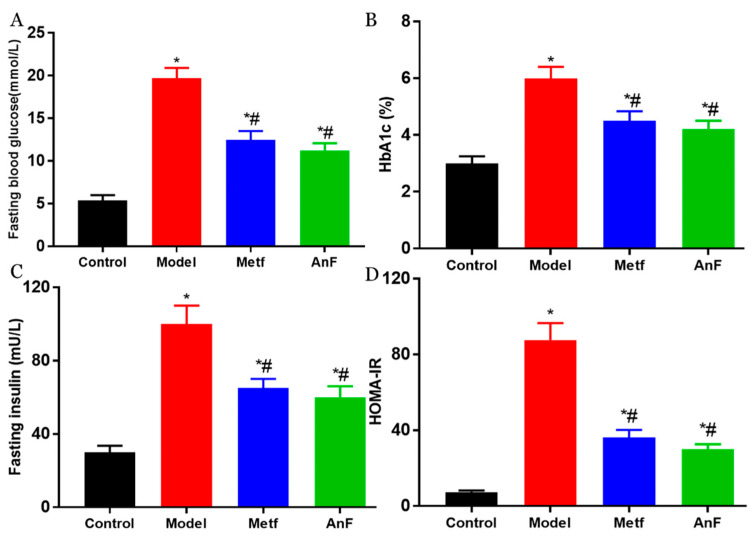
Effects of AnF on glucose-insulin homeostasis in db/db mice. Fasting blood glucose (**A**); hemoglobin A1c (HbA1c) (**B**); Fasting insulin (**C**); homeostasis model assessment-insulin resistance (HOMAI-IR) (**D**). Control, C57BL/6J mice; Model, db/db mice; Metf, db/db mice with 200 mg/kg/d of metformin; AnF, db/db with 200 mg/kg/d AnF. Data are expressed as the mean ± SEM. * *p* < 0.05, compared with the Control group; # *p* < 0.05, compared with the Model group. Six mice of each group were analyzed.

DM is characterized by hyperglycemia and systemic insulin resistance [45]. Thus, we investigated the effect of AnF on glucose-insulin homeostasis in db/db mice. As shown in Figure 6A, AnF significantly alleviated fasting hyperglycemia compared with the Model group (*p* < 0.05). The administration of AnF as well as metformin, resulted in an effective decrease in hemoglobin A1c (HbA1c) level (*p* < 0.05) compared with the Model group (Figure 6B), which indicated that AnF had a long-term effect on alleviating hyperglycemia. As shown in Figure 6C,D, the results showed the significant effect of AnF on suppressing hyperinsulinemia and lowering the homeostasis model assessment-insulin resistance (HOMA-IR) index in db/db mice (*p* < 0.05). All of these analyses confirmed the glucose–insulin homeostasis effects of AnF in db/db mice.

### 2.8. Effects of AnF on GLP-1 Level in db/db Mice

**Figure 7:**
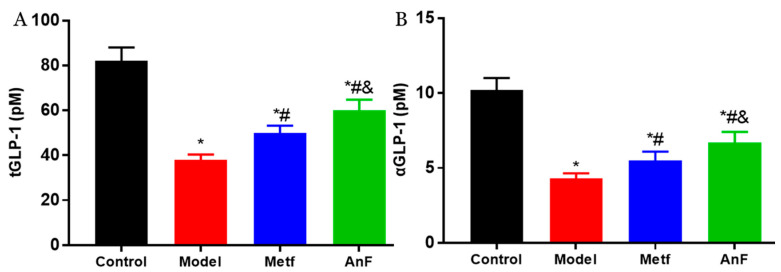
Effects of AnF on serum total glucagon-like peptide-1 (tGLP-1) and active GLP-1 (aGLP-1) levels in db/db mice. tGLP-1 (**A**) and aGLP-1 (**B**) levels. Control, C57BL/6J mice; Model, db/db mice; Metf, db/db mice with 200 mg/kg/d of metformin; AnF, db/db with 200 mg/kg/d AnF. Data are expressed as the mean ± SEM. * *p* < 0.05, compared with the Control group; # *p* < 0.05, compared with the Model group; and & *p* < 0.05, compared with the Metf group. Six mice of each group were analyzed.

Increasingly, studies have demonstrated that SGLT1 in the intestine serves as a sensor for the acute glucose-induced GLP-1 secretion [46,47]. The short-term inhibition of SGLT1 in the intestine can delay the absorption of glucose, and this unabsorbed glucose can stimulate L cells to secrete GLP-1 [48,49]. GLP-1 can act on pancreatic β cells to increase insulin release in a glucose-dependent manner and decrease pancreatic glucagon secretion, which both contribute to the antihyperglycemic effect [50]. Studies have indicated that the inhibition of SGLT1 in the intestine can not only increase the content of serum active GLP-1 (aGLP-1) level but also significantly increase the serum total GLP-1 (tGLP-1) level [51,52]. The effects of AnF on inhibiting postprandial blood glucose level by in vitro (Caco-2 monolayer model), semi-in vivo (everted gut sac model), and in vivo assays indicate that AnF can inhibit SGLT1 activity. Thus, we investigated the effect of AnF on the level of GLP-1 in db/db mice further. As shown in Figure 7, AnF effectively increased the serum content of tGLP-1 and aGLP-1 compared with the Model group, but this was still lower compared to the normal levels (*p* < 0.05), which indicated the moderate inhibition of SGLT1 by AnF can cause GLP-1 release without side effects on the digestive system.

### 2.9. Interaction Study between Fucoidans and SGLT1

**Figure 8:**
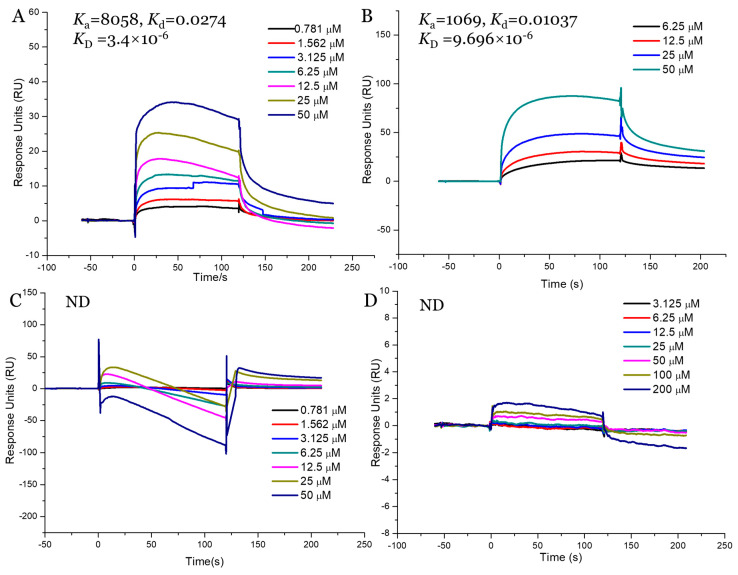
Surface plasmon resonance (SPR) kinetic binding analysis of the interactions of Na^+^/glucose cotransporter 1 (SGLT1) with AnF (**A**), FvF (**B**), KcF (**C**), and LjF (**D**). ND, not detected.

Our in vitro and in vivo assays demonstrated that AnF could effectively decrease the glucose transport and postprandial blood glucose levels, while LjF and KcF could not, which may have been due to the various inhibitions of SGLT1 activity by fucoidans. Thus, we validated the binding affinities between these three fucoidans (AnF, LjF, and KcF) and SGLT1 protein further using SPR. The response units were recorded in real-time as sensor grams using the BIAcore system (Figure 8), and the kinetic parameters (such as the binding constant (*K*_a_, M^−1^s^−1^), dissociation constant (*K*d, 1/s), and average dissociation constant (*K*_D_, M)) were also summarized. These results showed that AnF and FvF with type II structures bound directly to SGLT1, and the *K*_D_ was 3.4 × 10^−6^ M and 9.696 × 10^−6^ M, respectively. In contrast, no significant affinity was detected between KcF/LjF and SGLT1. Therefore, we concluded that the inhibitory effect of AnF on SGLT1 was due to the strong binding between them, which could effectively block the glucose transport capacity of SGLT1. As expected, AnF and FvF, with type II structures, bound directly to SGLT1, while KcF and LjF, with type I structures, could not, which was consistent with the effects of these respective molecules on glucose transport and postprandial blood glucose levels in vitro and in vivo assays and our previous study [30]. As SGLT1 is a crucial factor for regulating glucose transport and postprandial blood glucose levels, these data indicated that SGLT1 was probably a potential target for fucoidans with type II structure to exert the hypoglycemic effects.

## 3. Discussion

Postprandial hyperglycemia is a key factor in the formation and development of DM, and abnormally elevated intestinal SGLT1 activity is the main cause in DM patients with postprandial hyperglycemia. As SGLT1 is crucial for intestinal glucose absorption, important strategies in the prevention and treatment of hyperglycemia include exploring compounds with significantly inhibitory effects on SGLT1 activity [53]. In our previous studies, we found that fucoidan with a type II structure exhibited significant inhibition of α-glucosidase activity in vitro, rather than fucoidan with a type I structure [31]. In addition, AnF reduced α-glucosidase activity to about 30%, whereas the application of FvF resulted in a 90% decrease in α-glucosidase activity relative to that of the control. And we also elucidated that FvF increased glucose consumption and relieved insulin resistance via ROS-mediated JNK and Akt signaling pathways by using HepG2 cell line. In vivo, FvF could regulate lipid metabolism to attenuate metabolic syndrome [30]. For clarifying the mechanism of inhibiting glucose absorption of type II structure fucoidan, another type II fucoidan, AnF with lower α-glucosidase inhibition was used to rule out other factors. Furthermore, it is encouraging to develop potential antihyperglycemic polysaccharide compounds from natural resources. Thus, we assessed the effects of AnF (type II structure), LjF (type I structure), and KcF (type I structure) on postprandial blood glucose levels, and found that only AnF could effectively alleviate postprandial hyperglycemia. The underlying mechanism might be that only AnF and FvF, with type II structures, exhibited effective binding affinity to SGLT1 via SPR, which further indicated that fucoidans with type II structures could reduce postprandial hyperglycemia by suppression of SGLT1 activity.

SGLT1 is pivotal for the absorption of glucose in the intestinal tract [54]. The inhibitory activities of fucoidans on SGLT1 activity were evaluated by in vitro (Caco-2 monolayer model), semi-in vivo (everted gut sac model), and in vivo (OGTT in Kunming mice) assays, which demonstrated that only AnF could significantly decrease the transport of glucose by inhibiting the activity of SGLT1, while LjF and KcF could not. It has been verified that the bioactivity of fucoidans depends highly on their structural properties [21]. Both LjF and KcF had no marked effects on glucose transport, which indicated that MW and fucose content may not play a pivotal role in this ability. In addition, the various effects of AnF and LjF on glucose transport indicated that the type of fucosidic linkages may play a more crucial role. Combined with the result that FvF with a type II structure could conspicuously reduce the transport of glucose, these results indicate the importance of the type of glycosidic linkages in inhibiting glucose transport.

It was also reported that type II fucoidans could be a promising α-glucosidase inhibitor to reduce blood glucose levels [31]. Thus, OGTT was used to evaluate the effects of fucoidans on SGLT1 activity in vivo, avoiding the influence of α-glucosidase, and the absorption of glucose in the intestinal tract can only be realized through the transport of the SGLT1 protein. OGTT results in Kunming mice and db/db mice further confirmed the effect of AnF on decreasing postprandial blood glucose, improving glucose tolerance and insulin sensitivity. In addition, AnF with a type II structure effectively increased the GLP-1 levels, which further confirmed the inhibition of AnF on SGLT1 [51,52]. Moreover, fucoidans cannot be digested by gastric enzymes in the gastrointestinal tract and exhibit extremely low bioavailability after oral administration [43], which indicated that fucoidans, with type II structures, can play an effective and lasting role in inhibiting SGLT1 activity in vivo. Additionally, it has been reported that the in vivo effect of fucoidan on blood coagulation was not obvious, probably due to its low intestinal absorption [55]. However, it is necessary to assess the cytotoxicity and blood-thinning properties (e.g., activated partial thromboplastin time (APTT)) of various fucoidans for developing as potential nutraceuticals or dietary supplements for treating the hyperglycemia. All in all, we will pay more attention to the toxicity of various fucoidans in future studies.

## 4. Materials and Methods

### 4.1. Preparation of Fucoidans

Three different brown seaweeds, *Ascophyllum nodosum* (collected from the Irish Sea), *Laminaria Japonica* (collected from the South China Sea), and *Kjellmaniella crassifolia* (collected from the South China Sea), were purchased from Kunshan Yihong Seaweed Co. Ltd. (Kunshan, Jiangsu, China). The extraction and purification processes of AnF, LjF, and KcF were carried out according to the method, as previously described [30,56]. Briefly, brown algae were dried, powdered, and then pass through a 12-mesh net, followed by delipidating (60 °C, 4 h, 95% ethanol, 1:20) and two cycles of extraction with double-distilled water (ddH_2_O) (80 °C, 3 h, 1:20). After centrifugation at 5000 rpm for 10 min, the supernatants were combined and concentrated, and then anhydrous ethanol was added to achieve a final concentration of 80%, and the supernatants were left overnight at 4 °C. After centrifugation at 5000 rpm for 10 min, the crude fucoidans were obtained. Then, the precipitate was redissolved in ddH_2_O, and 3 M of CaCl_2_ solution was added to remove alginate completely until no precipitation occurred. The solution was centrifuged at 8000 rpm for 10 min to remove precipitates, and the supernatant containing fucoidan was dialyzed in a dialysis bag with a 7000 Da MW cutoff, then lyophilized to obtain the refined fucoidans. Chemicals reagents were obtained from the Sigma Chemical Co. (Sigma–Aldrich, St. Louis, MO, USA) unless otherwise stated.

### 4.2. Physicochemical Properties Analysis of Fucoidans

The sulfate content was determined by the BaCl_2_-gelatin method as follows [57]. Fucoidan (3 mg/mL) was degraded in 1 M of HCl at 110 °C for 6 h, then the absorbance of the degraded solution was determined at 400 nm after mixing with an equal volume of BaCl_2_-gelatin. The sulfate content was calculated based on a standard curve, which was established with a Na_2_SO_4_ standard. The monosaccharide composition was analyzed with the acid hydrolysis method described previously [58]. In brief, the monosaccharide composition was determined using a1-phenyl-3-methyl-5-pyrazolone precolumn derivatization HPLC on an Agilent Eclipse XDB-C18 Column (Agilent, Santa Clara, CA, USA). Sample (10 μL) was eluted with 0.1 mol/L phosphate buffer (pH 6.7) and acetonitrile (83:17 volume fraction) at a flow rate of 1 mL/min at 30 °C. Then, a UV detector was used to detect the signal at 245 nm. MW was determined using high-performance gel permeation chromatography coupled with a multi-angle laser light scattering instrument. The MWs of fucoidans were determined using an Agilent 1260 HPLC system (Agilent Technologies, CA, USA) on the Shodex Ohpak SB-HQ 804 column in series with an SB-HQ 803 column (TosoHaas Corp., Tosoh, Japan) detected with a Wyatt Dawn Heleos II multi-angle laser scattering system (Wyatt Technology, Santa Barbara, CA, USA) and refractive index detector. One hundred microliters of sample was eluted with 0.1 M of Na_2_SO_4_ solution at a flow rate of 0.6 mL/min at 35 °C. MWs were calculated using Astra 5.3.4.20 software (Wyatt Technology, Santa Barbara, CA, USA). The type of glycosidic linkages of fucoidans was determined by the published papers, i.e., FvF (31), AnF (32), KcF (33), and LjF (34).

### 4.3. Effects of Fucoidans on Glucose Absorption Using Caco-2 Monolayer Model

The inhibitory effects of fucoidans on glucose uptake in vitro were measured using a human colon cancer cell line monolayer model (Caco-2 cells, from the Cell Bank of the Chinese Academy of Sciences, Shanghai, China) incubated with 2-NBDG (MedChem Express, Monmouth, NJ, USA) in various conditions. Caco-2 cells were cultured in Dulbecco’s Modified Eagle Medium (DMEM) with 10% FBS, 1% penicillin/streptomycin, 25 mM HEPES, and 0.35 g/L sodium bicarbonate (Gibco, Carlsbad, CA, USA) at 37 °C with 5% CO_2_. A Caco-2 monolayer model was established as follows [59]. Briefly, Caco-2 cells were adjusted to 2 × 10^5^ cells/mL, and 100 μL of this cell suspension was inoculated in the upper layer of a transwell compartment (0.4 μm, 1.12 cm^2^, PET) (Corning Inc., Corning, NY, USA) and incubated at 37 °C for 2 min. Then, 500 μL of DMEM was added to the upper layer, while 1.5 mL DMEM was added to the lower layer for Caco-2 cells to differentiate into enterocyte-like cells at 37 °C. Then, the TEER was measured, which could evaluate the integrity of the Caco-2 monolayer cells. In addition, another index to evaluate the successful construction of the Caco-2 monolayer model was to determine the ALP activity ratio of the apical side to the basolateral side. In the Caco-2 monolayer cell model, the apical side located to the upper side of the transwell had higher ALP activity, while the basolateral side located to the lower side had lower ALP activity. The enzyme activities of both sides of the transwell were measured using the ALP ELISA kit (Shanghai Enzyme-linked Biotechnology Co. Ltd., Shanghai, China) according to the manufacturer’s recommended protocol on the 4th, 8th, 12th, 16th, and 21st-day post-induction, respectively. Then, the ALP activity ratio was calculated. The 2-NBDG uptake in the Caco-2 monolayer model was conducted as previously described [60]. Briefly, the culture medium was removed from each well and replaced with 100 μL of HBSS buffer in the presence of 2-NBDG (100 μM) or 2-NBDG (100 μM) together with 400 μg/mL of specific fucoidan. Then the cells were incubated at 37 °C for 30 min. Finally, the fluorescence intensity (Ex/Em = 485/535 nm) in the lower layer was measured using a Spark 10M (Tecan Trading AG, Männedorf, Switzerland).

### 4.4. Effects of Fucoidans on OGTT in Kunming Mice and Glucose Transport Using Everted Gut Sac Model

An everted gut sac model was established as previously described [40]. Briefly, six-week-old male Kunming mice were purchased from the Vital River Laboratory Animal Technology Co. Ltd. (Beijing, China). The mice were raised in ventilated cages, maintained in a light-dark cycle of 12 h at 23 °C–25 °C, with free access to water and food. After a two-week adaptive period, the mice were randomly divided into four groups of six each. OGTT was performed as follows [61,62]. The mice of each group were fasted for 15 h, then the experimental groups were given the specific fucoidan by gavage at a dose of 200 mg/kg, while the Control group was given the same volume of saline. Then, mice were given a 20% glucose solution at a dose of 2 g/kg by oral gavage in 15 min. Next, blood glucose levels were detected using a standard glucometer (Johnson & Johnson, New Brunswick, NJ, USA) at 0, 30, 60, 90, and 120 min by cutting the tail tip. In addition, the increment of plasma glucose following glucose loading was expressed in terms of the area under curve, using the trapezoidal rule. The jejunum was separated from the Kunming mice one week after the OGTT experiment. Mice were euthanized by pentobarbital sodium injection (80 mg/kg) accompanied by isoflurane inhalation to maintain anesthesia. The jejunum was cut into 5-cm segments and quickly transferred into cold Krebs–Ringer buffer in the state of oxygen maintenance. Due to the overturning of the intestinal sac, SGLT1 protein originally on the serosal side moved to the outside, so the intestinal epithelial cells could absorb glucose from the outside to the inside in. Thus, Krebs–Ringer buffer was injected into the intestinal capsule of the everted gut sac model, then placed in Krebs–Ringer buffer (containing 30 mM D-glucose) with various concentrations of specific fucoidan in the experimental groups, while the group treated with Krebs–Ringer buffer (containing 30 mM D-glucose) was used as the Control. After incubation for 30 min at 37 °C, the glucose concentrations of the inside and outside of the intestinal capsule were determined using the glucose oxidase-peroxidase method with a glucose oxidase kit (Applygen Technologies Inc., Beijing, China) [63]. Additionally, the glucose intake ratio was calculated by the glucose concentration of the inside, divided by the glucose concentration of the outside. All animal procedures were approved by the Committee of Experimental Animals of School of Medicine and Pharmacy, Ocean University of China (OUCSMP-18081201), and conformed to the Guide for the Care and Use of Laboratory Animals published by the United States National Institutes of Health (NIH Publication No 85-23, revised 1996).

### 4.5. Effects of AnF on Alleviating Hyperglycemia in db/db Mice

Briefly, eight-week-old male db/db mice were provided by the Model Animal Research Center of Nanjing University (Nanjing, China). In addition, eight-week-old male C57BL/6J mice were purchased from the Vital River Laboratory Animal Technology Co. Ltd. (Beijing, China) as a Control group. The mice were raised as described in the methods in Section 4.4. After a two-week acclimation period, the db/db mice were randomly divided into three groups as follows, with six mice in each group: the Metf and AnF groups received either metformin (200 mg/kg/d dissolved in saline) or AnF (200 mg/kg/d dissolved in saline) by gavage for four weeks, while the Model group was given an equal amount of saline. Body weights were measured every week. OGTT was conducted at the end of the trial as follows: mice were fasted for 15 h, and the fasting blood glucose levels were detected. Then, the mice were given a 20% glucose solution by gavage at a dose of 2 g/kg body weight. The changes in blood glucose levels were detected, as described in Section 4.4. All animal procedures were approved by the Committee of Experimental Animals of School of Medicine and Pharmacy, Ocean University of China (OUCSMP-18081201), and conformed to the Guide for the Care and Use of Laboratory Animals published by the United States National Institutes of Health (NIH Publication No 85-23, revised 1996).

### 4.6. Effects of AnF on Biochemical Indexes in db/db Mice

Db/db mice with various treatments were finally euthanized by pentobarbital sodium injection (80 mg/kg) accompanied by isoflurane inhalation to maintain anesthesia after being fasted for 15 h. Blood samples were collected via retro-orbital bleeding, then centrifuged at 2000× *g* for 15 min to obtain serum for serological assays. The levels of fasting insulin and HbA1c in serum were determined using a mouse insulin ELISA kit and a mouse HbA1c ELISA kit from Omnimabs (Alhambra, CA, USA) according to the manufacturer’s instructions, respectively. HOMA-IR was calculated as fasting insulin (mU/L) × fasting glucose (mM)/22.5. For tGLP-1 and aGLP-1 contents detection, a DPP-4 inhibitor was quickly added into the blood samples and mixed evenly. Then, the blood was centrifuged for 10 min at 2000× *g* to obtain the supernatant for assays. The contents of tGLP-1 and aGLP-1 were determined using mouse ELISA kits (Linco, St. Charles, MO, USA) according to the manufacturer’s instructions, respectively.

### 4.7. Binding Kinetics Analysis of Interaction between SGLT1 and Fucoidans

The binding kinetics between various fucoidans and SGLT1 protein was determined by an SPR biomacromolecule interaction analyzer BIAcore T200 (General Electric Company, Boston, MA, USA), as previously described [64,65]. After washing the surface of the CM5 chip (General Electric Company, Boston, MA, USA) with PBS-P running buffer (General Electric Company, Boston, MA, USA), the surface of the chip was activated with 0.4 M EDC/0.1 M NHS for 420 s at a flow rate of 10 μL/min. Immediately after activation, an SGLT1 solution (20 μg/mL) (ab152683, Abcam, Cambridge, UK) in sodium acetate buffer (pH 4.5) was added onto the chip surface for 30 s at a flow rate of 10 μL/min. After that, the chip surface was sealed by incubating with 1 M of ethanolamine (pH = 8.5) for 30 min. PBS-P buffer was running for at least 2 h to stabilize the baseline. To assess the real-time binding of fucoidans to SGLT1, varying concentrations of specific fucoidan were injected over the sensor chip surface at a flow-rate of 30 μL/min for 120 s, followed by another 900 s dissociation period. The sensor surface was regenerated by 0.1 mM NaOH for 10 s. The response was monitored as a function of time (sensor gram) at 25 °C and subtracted from the response of the reference surface. The binding constant (*K*a), dissociation constant (*K*_d_), and average dissociation constant (*K*_D_ = *K*_d_/*K*a) of the interaction between various fucoidans and SGLT1 protein could be calculated from curve fitting. Kinetic parameters were evaluated using the BIAcore T200 evaluation software 3.1 (General Electric Company, Boston, MA, USA).

### 4.8. Statistical Analysis

Data are presented as the mean ± standard error of the mean (SEM). The difference between groups was analyzed using SPSS software (v.20.0; IBM, Armonk, NY, USA) via one-way ANOVA with Student’s *t*-test. And, Tukey’s honest significant difference test was used for the analysis of multiple comparisons. Difference was considered to be statistically significant between various groups when *p* < 0.05. The results were interpreted using GraphPad Prism software (v.7.0; GraphPad Software Inc., San Diego, CA, USA).

## 5. Conclusions

For the first time, in vitro (Caco-2 monolayer and SPR assay), semi-in vivo (everted gut sac), and in vivo (Kunming mice and db/db mice) models were used to evaluate the effects of various fucoidans on suppressing hypoglycemia, especially postprandial hyperglycemia. Our data indicated the potential effects of AnF on the regulation of blood glucose levels by direct inhibition of glucose transport via SGLT1, therefore, remarkably reducing glucose transport and relieving postprandial hyperglycemia. In conclusion, fucoidans with type II structures (such as FvF and AnF) have the potential to be a promising candidate compound in the treatment of postprandial hyperglycemia via its direct binding to SGLT1 and inhibition of its glucose transport activity, while KcF and LjF with type I structures cannot. Our study demonstrates that the type of glycosidic linkages of fucoidans may play a more crucial role in their hypoglycemic effects via inhibiting SGLT1 activity. However, further research is still necessary to clarify the exact structure–activity relationship of fucoidans as SGLT1 inhibitors and the precise molecular binding mode between fucoidans and SGLT1 protein.

## Figures and Tables

**Table 1:** Compositions of fucoidans from various sources.

Sample	Source	Linkage Mode	SO_4_^2−^ (%)	MW (kDa)	Monosaccharide Composition (%)							
					Man	GlcN	Rha	GlcA	Glc	Gal	Xyl	Fuc
AnF	*Ascophyllum nodosum*	1→3/1→4	22.7	210	7.5	0.6	0.3	3.3	8.0	3.8	17.9	58.5
LjF	*Laminaria japonica*	1→3	26.0	200	9.3	2.0	3.9	4.1	5.1	19.6	4.1	52.0
KcF	*Kjellmaniella crassifolia*	1→3	27.6	940	11.0	1.4	0.8	6.3	1.7	3.9	2.9	72.1
FvF [31]	*Fucus vesiculosus*	1→3/1→4	26.3	1039	ND	ND	ND	ND	1.0	1.9	2.3	94.8

AnF: fucoidan from *Ascophyllum nodosum*; LjF: fucoidan from *Laminaria japonica*; KcF: fucoidan from *Kjellmaniella crassifolia*; FvF: fucoidan from *Fucus vesiculosus*; MW, molecular weight; Man, mannose; GlcN, glucosamine; Rha, rhamnose; GlcA, glucuronic acid; Glc, glucose; Gal, galactose; Xyl, xylose; Fuc, fucose.

## Abbreviations

DM: diabetes mellitus; SGLT1: Na^+^/glucose cotransporter 1; OGTT: oral glucose tolerance test; AnF: fucoidan from *Ascophyllum nodosum*; KcF: fucoidan from *Kjellmaniella crassifolia*; LjF: fucoidan from *Laminaria japonica*; 2-NBDG: 2-Deoxy-2-[(7-nitro-2,1,3-benzoxadiazol-4-yl) amino]-D-glucose; SPR: surface plasmon resonance; ALP: alkaline phosphatase; GLP-1: glucagon-like peptide 1; DPP-4: dipeptidyl peptidase 4; aGLP-1: active GLP-1; tGLP-1: total GLP-1; HOMA-IR: homeostasis model assessment-insulin resistance; HbA1c: hemoglobin A1c; ddH2O: double-distilled water; DMEM: Dulbecco’s Modified Eagle Medium; TEER: transepithelial electrical resistance; MEM: minimum Eagle’s medium; BSA: bovine serum albumin; FvF: fucoidan from *Fucus vesiculosus*, MW: molecular weight; UAs: uronic acids; DMEM: Dulbecco’s Modified Eagle Medium; Metf: metformin; db/db: leptin receptor-deficient; APTT: activated partial thromboplastin time; ROS: reactive oxygen species; JNK: c-Jun N-terminal kinase; Akt: protein kinase B.
